# Tracking transparent monogenean parasites on fish from infection to maturity

**DOI:** 10.1016/j.ijppaw.2015.06.002

**Published:** 2015-07-07

**Authors:** Alejandro Trujillo-González, Constantin C. Constantinoiu, Richard Rowe, Kate S. Hutson

**Affiliations:** a Marine Parasitology Laboratory, Centre for Sustainable Tropical Fisheries and Aquaculture, College of Marine and Environmental Sciences, James Cook University, Townsville, Queensland, Australia; b Centre for Biosecurity in Tropical Infectious Diseases, College of Public Health, Medical and Vet Sciences, James Cook University, Townsville, Queensland, Australia; c College of Marine and Environmental Sciences, James Cook University, Townsville, Queensland, Australia

**Keywords:** Monogenea, *Neobenedenia*, Capsalidae, Development, Migration, Microhabitat, Fluorescent labelling

## Abstract

The infection dynamics and distribution of the ectoparasitic fish monogenean *Neobenedenia* sp. (Monogenea: Capsalidae) throughout its development was examined on barramundi, *Lates calcarifer* (Bloch) (Latidae), by labelling transparent, ciliated larvae (oncomiracidia) with a fluorescent dye. Replicate fish were each exposed to approximately 50 fluorescent oncomiracidia and then examined for parasites using an epifluorescence stereomicroscope at 10 time intervals post-exposure (15, 30, 60, 120 min, 24, 48 h, four, eight, 12, and 16 days). Fluorescent labelling revealed that parasites attached underneath and on the surface of the scales of host fish. Parasite infection success was 20% within 15 min, and peaked at 93% two days post-exposure, before gradually declining between four and sixteen days. Differences in parasite distribution on *L. calcarifer* over time provided strong evidence that *Neobenedenia* sp. larvae settled opportunistically and then migrated to specific microhabitats. Parasites initially attached (<24 h) in greater mean numbers on the body surface (13 ± 1.5) compared to the fins (4 ± 0.42) and head region (2 ± 0.41). Once larvae recruitment had ceased (48 h), there were significantly higher mean post-larvae counts on the head (5 ± 3.4) and fins (12 ± 3) compared to previous time intervals. *Neobenedenia* sp. aggregated on the eyes, fins, and dorsal and ventral extremities on the main body. As parasites neared sexual maturity, there was a marked aggregation on the fins (22 ± 2.35) compared to the head (4 ± 0.97) and body (9 ± 1.33), indicating that *Neobenedenia* sp. may form mating aggregations.

## Highlights

• Movement of transparent fish monogeneans were tracked using a fluorescent dye.
• Parasites attach beneath scales of fish, which may affect treatment efficacy.
• Parasite larvae settled randomly and migrated to preferred microhabitats on the host.
• The eyes, fins, upper and ventral body surfaces were preferred by the parasite.
• *Neobenedenia* sp. aggregated on the fins prior to sexual maturity.

## Graphical abstract

**Figure:**
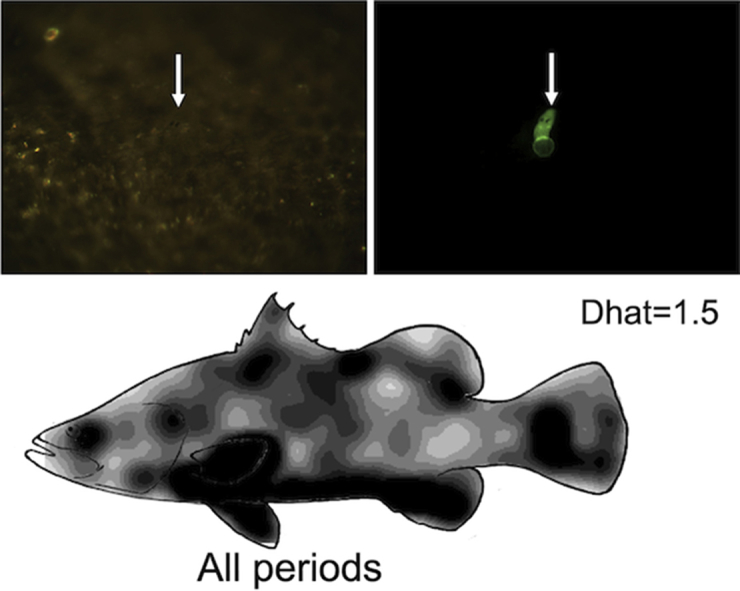

## 1 Introduction

The distribution of ectoparasites on their hosts is linked to adaptive strategies and life traits inherent to their evolution (Rohde, 2005). Parasite distribution and site-specificity have been associated with particular feeding guilds and diets (Marcogliese, 2002; Karvonen et al., 2007), mate finding habits (Chisholm et al., 1997; Chigazaki et al., 2000; Whittington and Ernst, 2002), evasion of the host immune system and toxic compounds (Buchmann and Linderstrøm, 2002; Sitjà-Bobadilla, 2008), camouflage and evasion of predators (Whittington, 1996), and avoidance of intra and interspecific competition (Rohde et al., 1995). Many ectoparasitic monogeneans are able to migrate over the body surface of their host and gain access to select microhabitats which are subsequently colonised (Cone and Burt, 1981; Whittington and Ernst, 2002) and where sexual maturity is reached (Kearn, 1984; Kearn and Whittington, 1992; Whittington and Ernst, 2002). Consequently, some host microhabitats exhibit greater ectoparasite loads and have increased susceptibility to epidermal damage and subsequent secondary infection (Kaneko et al., 1988; Thoney and Hargis, 1991; Buchmann and Linderstrøm, 2002; Leong and Colorni, 2002).

Capsalid monogeneans are harmful ectoparasites of ornamental and farmed fishes in tropical and subtropical marine environments (Thoney and Hargis, 1991; Deveney et al., 2001; Hirazawa et al., 2011; Hutson et al., 2012; Whittington, 2012). Within this group, *Neobenedenia* is comprised of particularly virulent species that exhibit low host specificity, a direct life cycle, high fecundity and environmentally resilient eggs (Bullard et al., 2000; Whittington, 2004; Ogawa et al., 2006; Militz et al., 2013; Dinh Hoai and Hutson, 2014). *Neobenedenia* spp. have been observed attached to all external surfaces of the host including the nostrils, eyes, mouth cavity and fins (Whittington, 1996; Ogawa et al., 2006; Hirazawa et al., 2011; Trujillo-González et al., 2014). The invasion route and site-selection of *Neobenedenia girellae* (Hargis) (see Whittington and Horton (1996) for an account of its likely synonymy with *Neobenedenia melleni*) has been previously described on Japanese flounder, *Paralichthys olivaceus* (Temminck and Schlegel) (see Bondad-Reantaso et al., 1995) and quantified on amberjack, *Seriola dumerili* (Risso) (see Hirayama et al., 2009). In both studies, post-larvae were found attached to the fins, while older parasites were found on the dorsal and ventral body surfaces. These studies used skin scrapings (Bondad-Reantaso et al., 1995) and stereomicroscopy (Hirayama et al., 2009) to detect live parasites.

The cryptic nature of *Neobenedenia* spp. makes live parasites extremely difficult to observe. Juveniles are small in size and may be transparent or have pigments that serve as camouflage when attached to the host (Whittington, 1996). Fluorescent labelling is a useful tool to examine the infection biology of parasites and has been previously used to describe the invasion route and site-selection of monogeneans (i.e. *Branchotenthes octohamatus* (Glennon, Chisholm and Whittington) on elasmobranchs (Glennon et al., 2007) and *Heterobothrium okamotoi* (Ogawa) on tiger puffer fish, *Takifugu rupribes* (Temminck and Schlegel) (see Chigazaki et al., 2000)) and actinospores in salmonid and cyprinid species (Yokoyama and Urawa, 1997). The aim of this study was to examine *Neobenedenia* sp. patterns of recruitment and parasite aggregation over a spatial–temporal scale on the body surface of barramundi, *Lates calcarifer* (Bloch). We used fluorescent labelling to examine monogenean distribution patterns over a prolonged period of time to account for potential differences in post-larval, juvenile and adult parasite distribution.

## 2 Materials and methods

### 2.1 Source of fish and *Neobenedenia* sp.

Fifty hatchery reared *L. calcarifer* (150 ± 30 *L*_*T*_ mm) were maintained in 100 L fresh water aquaria at the Marine Parasitology Laboratory, James Cook University. Fish had not been previously exposed to *Neobenedenia.* Fish were acclimated to sea water 24 h prior to experiments by increasing salinity to 10, 20, 30 and 35 ppt over 2 h intervals. Fish were fed until satiation every two days (∼1 g per fish) with pellets formulated for *L. calcarifer* (Ridley Aqua-Feed™). Parasite eggs were sourced from an experimental infection in the laboratory, which was established using methods previously described (Militz et al., 2013). *Neobenedenia* sp. investigated in this study is presently unidentified given the absence of diagnostic criteria to differentiate between geographical/host isolates and species (Whittington, 2004, 2012). Phylogenetic analysis of approximately 12 *Neobenedenia* spp. isolates collected from multiple fish hosts in northern Australia is ongoing and may provide species level-clarification (Brazenor, unpublished data). Meanwhile, representative specimens mounted on slides were accessioned in the South Australian Museum, Australia (SAMA) in the Australian Helminth Collection (AHC); SAMA AHC 35461 (see Hutson et al., 2012). Parasite eggs were collected daily and held in Petri dishes with fresh sea water. Newly hatched oncomiracidia (<3 h old) were gently aspirated with a pipette and used in the experiments described below.

### 2.2 Fluorescent labelling of *Neobenedenia* sp. oncomiracidia

*Neobenedenia* sp. oncomiracidia were labelled with a fluorescent marker to identify individual parasites on the fish body surface. A 10 mM stock solution of the fluorescent dye 5(6)-carboxyfluorescein diacetate N-succinimidyl ester (CFSE; Sigma–Aldrich, Castle Hill, NSW, Australia) was made by resuspending CFSE lyophilised powder in 100% dimethyl sulphoxide (DMSO), and stored at 4 ^°^C in dark conditions until use. The stock solution was diluted with filtered sea water (35 ppt) to produce a 30 nM working solution of CFSE for labelling (modified from Glennon et al., 2007). Approximately 400 *Neobenedenia* sp. oncomiracidia were held for 15 min in dark conditions in a 50 mL beaker with 25 mL of sea water (35 ppt) and 5 mL of 30 nM CFSE working solution. Only swimming oncomiracidia were selected for the experiments.

### 2.3 Neobenedenia infection *of L. calcarifer over time*

**Fig. 1:**
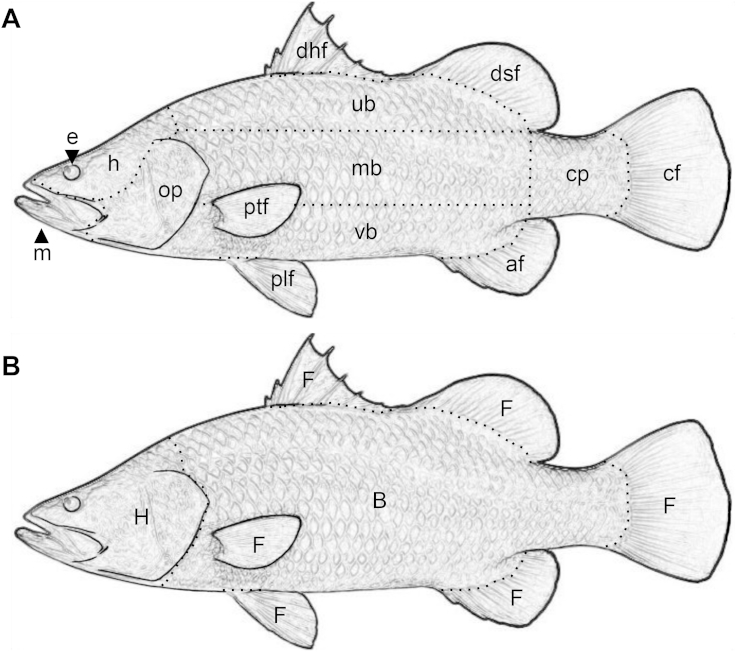
*Lates calcarifer* microhabitat terminology (A) and body surface regions (B) used for statistical analysis. af = anal fin; cf = caudal fin; cp = caudal peduncle; dhf = dorsal hard fin; dsf = dorsal soft fin; e = eye; h = head; m = mandible; mb = middle body; op = operculum; plf = pelvic fin; ptf = pectoral fin; ub = upper body; vb = ventral body. B = body; F = fins; H = head. Terminology is based on Helfman et al. (2009) and Roberts and Ellis (2012).

Fish were infected with fluorescent oncomiracidia and examined at 10 different time intervals to determine parasite distribution on the host body surface over its development. Fifty *L*. *calcarifer* were each infected with 50 ± 3 CFSE-labelled oncomiracidia, and held in individual aquaria (20 × 15 × 15 cm) in sea water (35 ppt; 25 ± 2.5 °C). A pilot study showed that parasite sampling and detection on the fish body surface took an average of 30 min for each individual fish. Thus, to enable precisely timed sampling, fish were infected over the course of five days, with ten randomly selected fish infected with labelled oncomiracidia each day. Each of the ten fish corresponded to one of ten time periods (15, 30, 60, 120 min, 24, 48, 96 h, eight, 12 and 16 d post-infection). Five replicates were made for each time period. Each fish was euthanised with a dose of Aqui-S aquatic anaesthetic (25 mL L^−1^ for 15 min), which does not cause parasite detachment (Sharp et al., 2004; Trujillo-González et al., 2014). Immediately following euthanasia, each fish was placed under an epifluorescence stereomicroscope (Olympus BX51) and both sides of the body surface (alternating left hand side first) were carefully examined for live parasites (Fig. 1A). The gills, buccal folds, buccal cavity and nasal chamber were not examined. Parasite location was recorded using an XY coordinate system based on a gridded translucent sheet of plastic (25 dots/cm^2^) placed over the fish. The tip of the mandible of each fish was placed on a marked location on the translucent grid to maintain a consistent coordinate origin. Scaled photographs were taken of each fish and of representative parasites attached to fish in each time period.

### 2.4 Statistical analysis

Infection success (total number of parasites on the host divided by the initial number of larvae introduced; Bush et al., 1997) was reported as a percentage and differences between time intervals were examined using a one-way ANOVA and a Tukey's HSD test in S-Plus 8.2. Parasite distribution on *L. calcarifer* was examined for complete spatial randomness (CSR) using R 3.1.0 for Windows. Parasites found on both sides of the fish were combined and parasites found underneath the pectoral fins (n = 3) were excluded for two dimensional parasite distribution analyses (Fig. 1A). Two different functions were used to test for spatial randomness including 1) origin to point neighbour distances (Ghat) and 2) point to point neighbour distances (Fhat). A complete spatial randomness simulation was then created based on a Monte Carlo test (Dhat), where Dhat = Ghat - Fhat. These three functions are used to test the assumptions of CSR (Diggle, 1983; Rowlingson and Diggle, 1993; Venables and Ripley, 2002). CSR was analysed using the “splancs” library in R 3.1.0 and Dhat values where ranked within 99 simulations of randomly distributed points. Complete spatial randomness was rejected when Dhat >90 (modified from Rowlingson and Diggle, 1993). A contour plot was created to illustrate *Neobenedenia* sp. distribution on the body surface of the host using a kernel density analysis with ARCGIS 10.1. Terminology used to describe the fish body surface microhabitats is defined in Fig. 1A.

Despite random allocation of fish to treatments, mean total length was higher in fish held in the eight and 12 day time periods (one-way ANOVA, F_9,38_ = 10.01 p < 0.05). To account for fish size, parasite density was analysed with a kernel spatial point analysis, using parasite coordinates to compare mean number of parasites per standardised unit of measure^2^. Parasite counts collected from fish at each time period (five fish per time period), were compared between three discrete regions on the fish: the head, the body and the fins (Fig. 1B). The number of parasites in each region was obtained by pooling parasite counts obtained from the coordinate data following the boundaries shown in Fig. 1B. In order to meet the assumptions of ANOVA a square root transformation was performed. Transformed mean parasite counts were compared between fish regions using one-way ANOVAs within each time period, and a two-way ANOVA to compare parasite counts between time periods with S-Plus 8.2.

## 3 Results

**Fig. 2:**
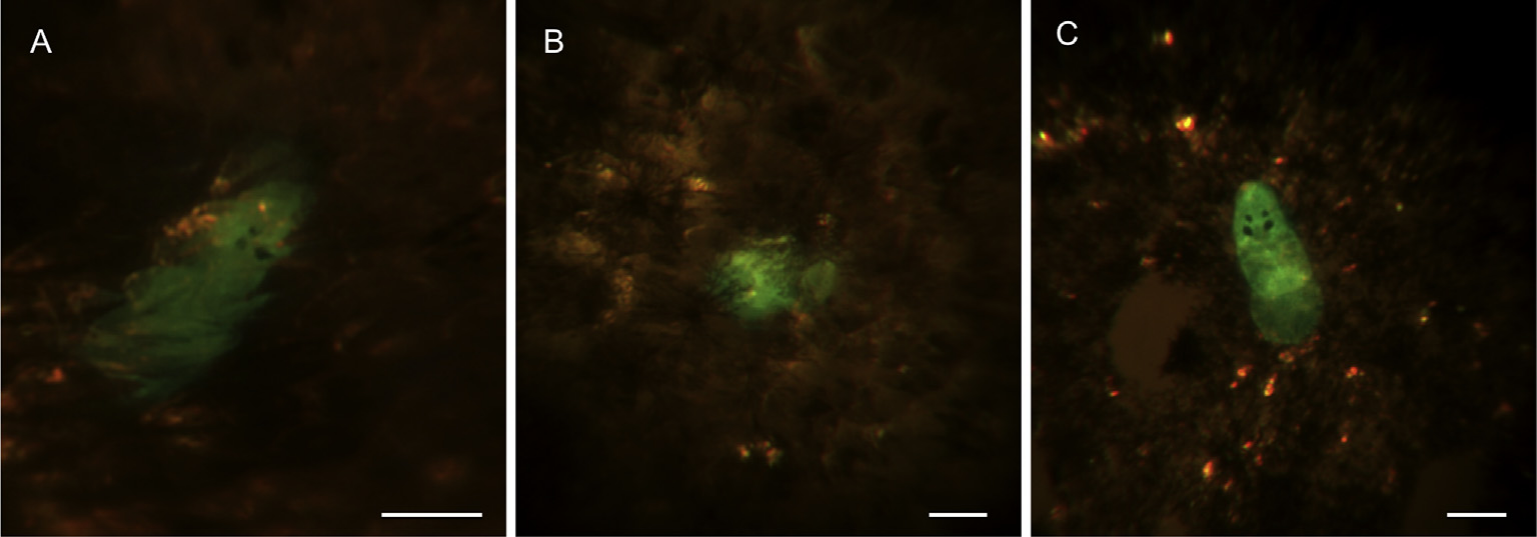
Live fluorescent *Neobenedenia* sp. juveniles attached beneath the scales of *Lates calcarifer* (A, B) and attached to the surface of the fish scales (C). Parasites are 1 h old (A, B) and 2 h old (C). Scale bar = 100 μm.

**Fig. 3:**
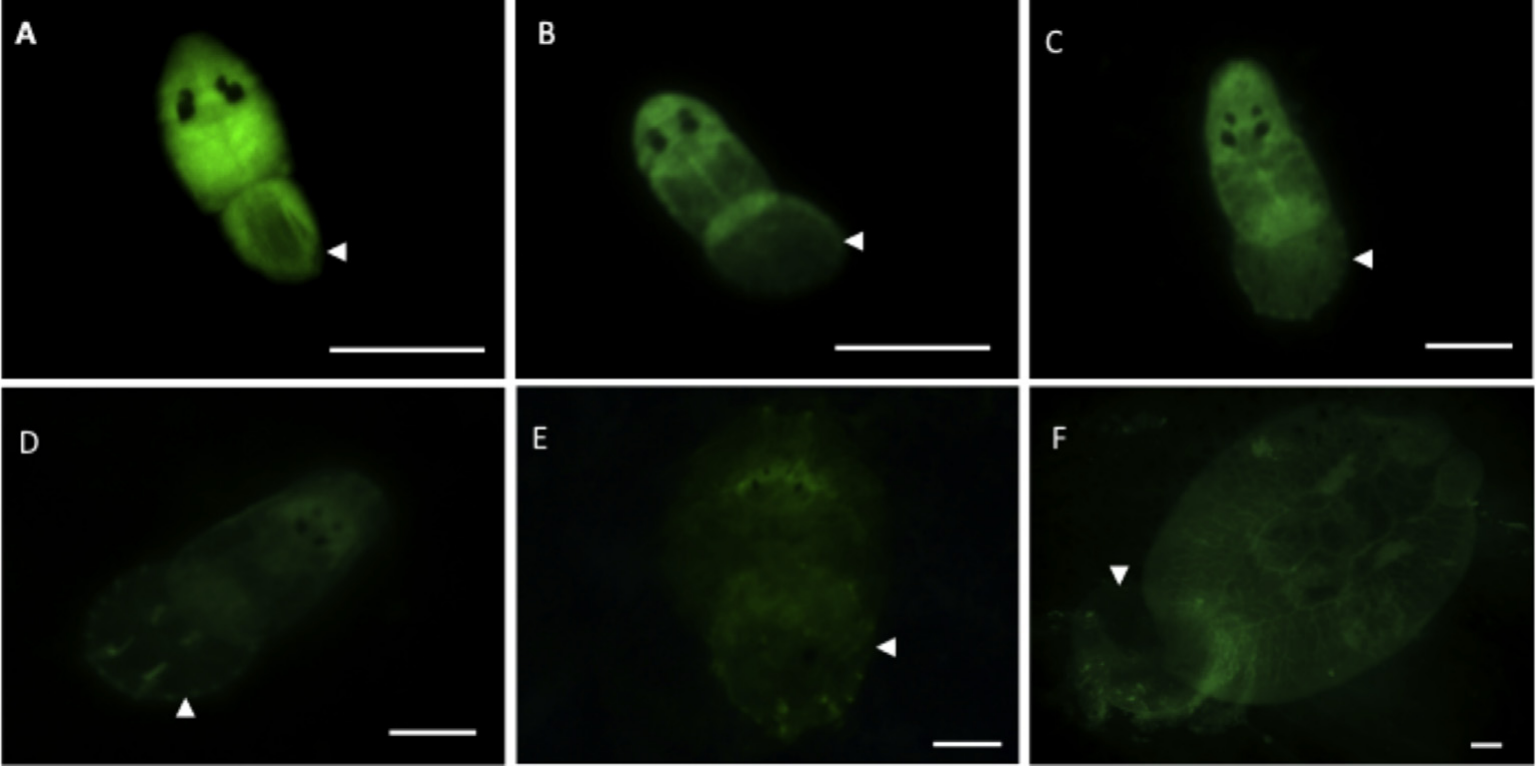
Live fluorescent *Neobenedenia* sp. attached to *Lates calcarifer* over time. Parasites observed attached to fish following 15 min (A), 30 min (B), 2 h (C), 48 h (D), 96 h (E) and 16 d (F) post-infection. Arrow shows the haptor of *Neobenedenia* sp. A slightly higher exposure was used when photographing parasites at 16 days post-infection to account for faded fluorescence. Scale bar = 100 μm.

The fluorescent marker enabled rapid and accurate inspection of the host body surface for the presence of small, newly settled post-larvae, including instances where parasites had lodged underneath fish scales (Fig. 2). The CFSE fluorescent signal emitted by the parasite was maintained throughout development, although the signal became weaker over time (Fig. 3). Parasites attached to the host using the haptor as an anchor point with the anterior end “tapping” the host's surface in the proximity of the parasite's total length. Parasites were occasionally observed to crawl over the body surface (as per Yoshinaga et al., 2000; Kearn, 2004).

**Fig. 4:**
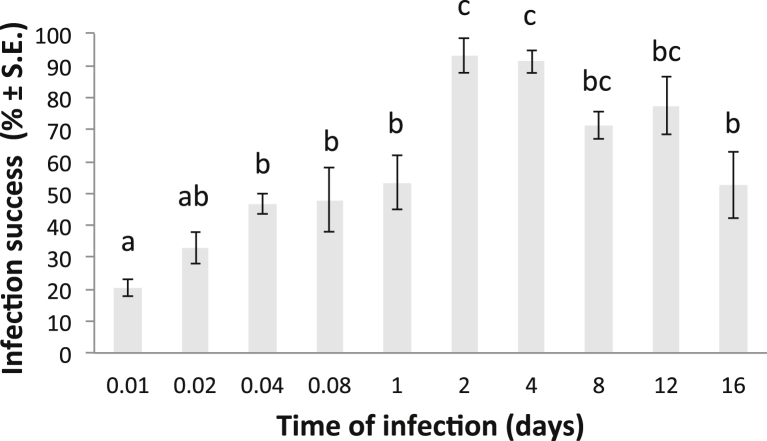
*Neobenedenia* sp. mean infection success on *Lates calcarifer* over time. ‘a’, ‘b’ and ‘c’ = differences between pairs of means determined using Tukey's HSD test, p < 0.05.

*Neobenedenia* sp. infection success increased through time, before gradually decreasing between day four and day 16. *Neobenedenia* sp. oncomiracidia used in this study live for an average of 37 ± 3 h in the absence of a host (at 35 ppt, 25 °C; Militz et al., 2013; Brazenor and Hutson, 2015). This indicates that the majority of viable oncomiracidia had successfully recruited to the host in the first 48 h of this study as shown by the peak in infection success (Fig. 4). Twenty ±2.5% of oncomiracidia had attached to the host within 15 min, and 32 ± 5%, 45 ± 3%, 45 ± 9% and 52 ± 9% attached by 30 min 1, 2 and 24 h, respectively (Fig. 4). Infection success peaked at 93%, two days post-exposure, before gradually decreasing in subsequent time intervals.

**Fig. 5:**
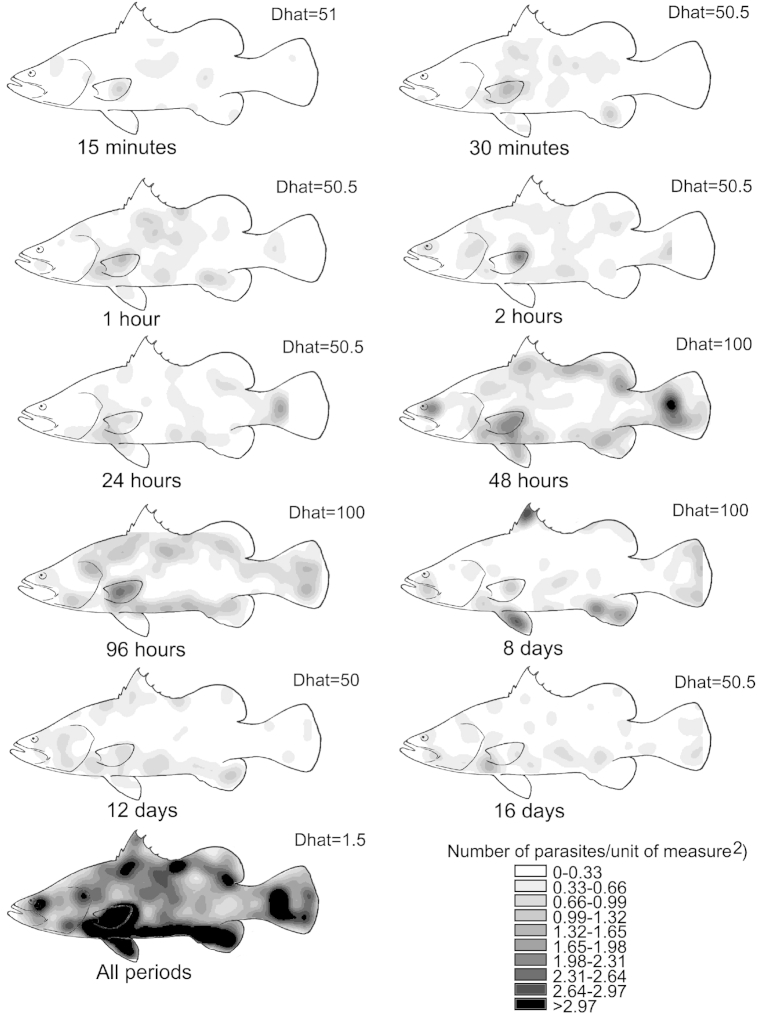
*Neobenedenia* sp. distribution on the body surface of *Lates calcarifer* over time. A kernel spatial point analysis was used to estimate the number of parasites/unit of measure^2^. Dhat values show the rank of the data within 99 simulations of randomly distributed points. Complete spatial randomness is rejected with values between 90 and 100.

Post-larvae randomly attached on the body surface of the host in the first 24 h (Fig. 5; Dhat<51). Parasites were aggregated between 48 h and 8 d post-exposure (Fig. 5; Dhat = 100), and exhibited a random distribution after 12 d post-exposure (Fig. 5; Dhat<51). Between 24 h and 8 days post-exposure there were fewer parasites on the middle body surface, and more on the fins, eyes, operculum, and on the peripheral region of the upper and ventral body surfaces of the host (Fig. 5). Between 12 and 16 days, parasites were concentrated on the head, ventral body surface and fins of the host (Fig. 5). Overall, higher numbers of parasites were observed on the eyes, fins and peripheral areas of the upper and ventral body surface compared to the head and middle body of *L. calcarifer* (Fig. 5, all periods).

**Fig. 6:**
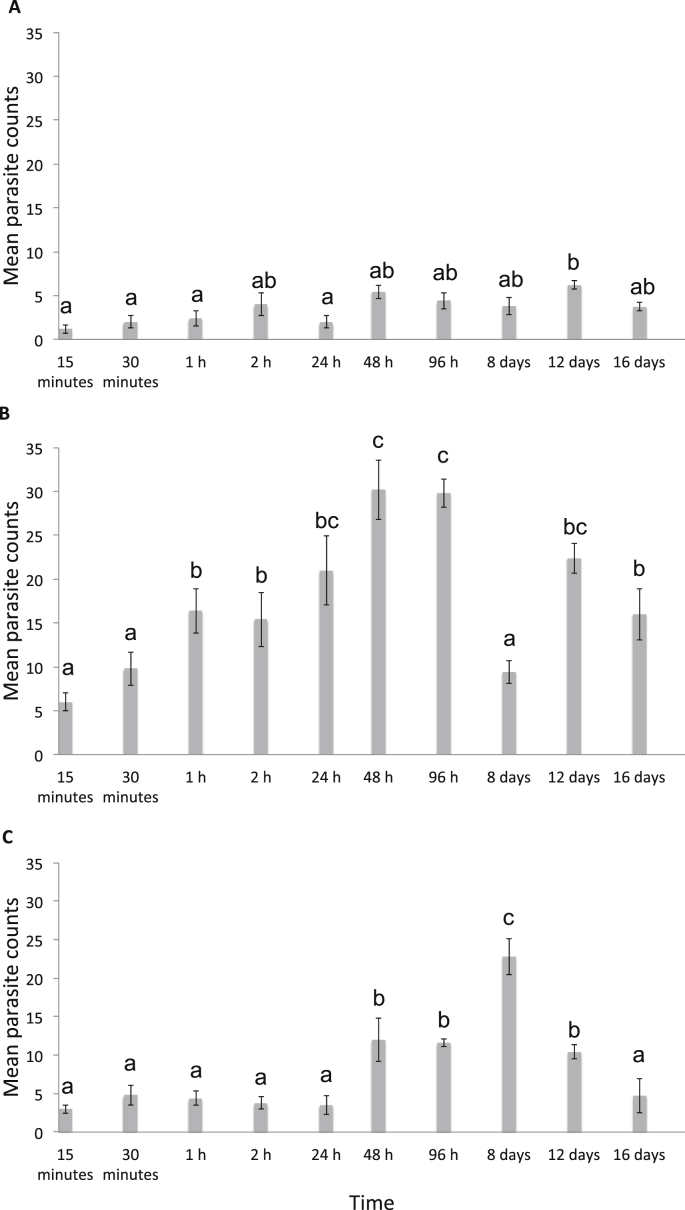
Mean parasite counts of *Neobenedenia* sp. infecting the head (A), body (B) and fins (C) of *Lates calcarifer* over time. ‘a’, ‘b’ and ‘c’ = differences between pairs of means determined using Tukey's HSD test.

Mean parasite counts were significantly higher on the body region compared to the head and fins of *L. calcarifer* in all time periods except day eight, where mean parasite counts were significantly higher on the fins (Fig. 6, one-way ANOVA, F2,12 = 34.29, p < 0.01). Mean parasite counts on the head and fins remained low over the first 24 h (Fig. 6A, C, two-way ANOVA, F18,114 = 10.02, p < 0.01), and gradually increased on the body of the host over the first 2 h of exposure (Fig. 6B). There was no significant difference in mean parasite counts within regions between 48 h and 96 h (Fig. 6). Parasite counts were significantly higher on the fins on day 8 (compared to all other time periods) and significantly lower on the body (compared to the five previous time periods) (Fig. 6, two-way ANOVA, F18,114 = 10.02, p < 0.01). Between day 12 and 16 mean parasite counts decreased in all regions (Fig. 6, two-way ANOVA, F18,114 = 10.02, p < 0.01).

## 4 Discussion

*Neobenedenia* sp. settled opportunistically before migrating to preferred microhabitats. In the first 24 h of infection, *Neobenedenia* sp. larvae exhibited a random distribution on the body surface of the host (<24 h, Fig. 5) which indicates that oncomiracidia may not be especially selective of their microhabitat during recruitment, but could be influenced by the need to find a host and ensure transmission (Kearn and Whittington, 1992; Whittington and Ernst, 2002). Considerable aggregation of parasites between 48 h and 8 days indicates that the majority of parasites migrated to specific microhabitats on the host following attachment (Fig. 5). No differences in mean parasite counts within regions between 48 and 96 h indicates that there was no considerable movement of parasites during this time (Figs. 5 and 6).

Random attachment of oncomiracidia, followed by migration of post-larvae to specific microhabitats, has been previously observed in monogeneans. Post-larvae of the gill parasite *Urocleidus adspectus* (Mueller) attach randomly on the body of yellow perch, *Perca flavescens* (Mitchill), prior to migration to the gills (Cone and Burt, 1981). *Entobdella soleae* (Lamarck) oncomiracidia attach on the upper surface of the common sole, *Solea solea* (Linnaeus), and migrate to the lower surface and posterior regions over time (Kearn, 1984). In the same manner, *Benedenia lutjani* (Whittington and Kearn) post-larvae attached to the body surface of the host and migrated to the pelvic fins (Whittington and Ernst, 2002). The random attachment of *Neobenedenia* sp. observed in this study differs to that previously observed for *N. girellae* (see Whittington and Horton (1996) for an account of its likely synonymy with *N*. *melleni*) where oncomiracidia settled predominantly on the fins of host fish species (i.e. *P. olivaceus* and *S. dumerili*) and then migrated to the main body surface as they grew (Bondad-Reantaso et al., 1995; Hirayama et al., 2009).

The fluorescent marker revealed that *Neobenedenia* sp. can attach underneath fish scales. This is a well-known microhabitat for transversotrematid trematodes (Cribb et al., 2002) but is a relatively rare occurrence, or is poorly documented, for monogeneans. Monogenean post-larvae of *U. adspectus* and juveniles and adults of *E. soleae* (Capsalidae) have been observed attached beneath the scales of their hosts (Cone and Burt, 1981; Kearn, 2004). In both studies, parasites attached to the underside of the scales with the haptor, with the anterior region, including the eye spots, uncovered (Cone and Burt, 1981; Kearn, 2004). The ability of *Neobenedenia* sp. to attach beneath the scales (Fig. 2) may have evolved in response to predation by cleaner organisms. Furthermore, this microhabitat may enable the parasite to be almost entirely secluded from the environment and could reduce the efficiency of current parasite management methods (e.g. chemical and freshwater bathing) in aquaculture.

*Neobenedenia* sp. was found in multiple microhabitats but parasites were more frequently found on the eyes, fins, dorsal and ventral body surface. This observation is in accordance with Hirazawa et al. (2011) who observed higher numbers of *N. girellae* (see Whittington and Horton, 1996) on the pelvic fins and body surface compared to the head of *S. dumerili*. Other monogeneans display high microhabitat specificity. For instance, some benedeniines live exclusively on specific fins or microhabitats on the head region such as lip folds and branchiostegal membranes (Whittington, 1996). Preference for the eyes, pelvic fins, dorsal and ventral body surfaces could confer adaptive benefits including avoidance of predation, competition and localised immune responses of the host (Whittington, 1996; Jones, 2001; Whittington and Ernst, 2002). The fins of the fish for example, could increase protection against predators and provide distinct feeding grounds or spatial resources for each developmental cohort (Whittington, 1996; Whittington and Ernst, 2002).

*Neobenedenia* sp. aggregated on the fins within 24 h of sexual maturity and exhibited a random distribution 12 d post-infection on the body of the host. *Neobenedenia* sp. reach sexual maturity (i.e. begin to lay eggs) on day nine post-infection in the described experimental conditions (i.e. 25 °C, 35 ppt; Brazenor and Hutson, 2015). Aggregation on the fins observed on day eight may be a result of parasites seeking other individuals for mating (Fig. 5 day 8; Fig. 6C). Migration to preferred microhabitats at the onset of mating has been observed for the monogenean, *B. lutjani*, where development of the reproductive organs corresponded with migratory movements on the host (Whittington and Ernst, 2002). Although *Neobenedenia* sp. can reproduce in isolation and do not necessarily need to cross-fertilise in order to produce viable offspring (Dinh Hoai and Hutson, 2014), migration to the fins at the onset of sexual maturity as a mating strategy could provide *Neobenedenia* sp. increased success of cross-insemination (Whittington and Kearn, 1993) or shared spermatophores between individuals (Ogawa et al., 2014). Aggregation on the fins may therefore confer advantages to find suitable mates and a random distribution after mating could be associated to *Neobenedenia* sp. egg production, its need to forage for resources (Whittington and Ernst, 2002), or a suitable location to disperse eggs (Whittington, 1996).

This study provides compelling evidence that ciliated *Neobenedenia* sp. larvae settled opportunistically and then migrated in search of specific microhabitats. Selected microhabitats included the eyes, fins, upper body and ventral body surfaces of the host. Reproduction could be an important factor determining *Neobenedenia* sp. distribution, indicated by parasites aggregating on the fins within 24 h of attaining sexual maturity. The fluorescent signal used in this study revealed that *Neobenedenia* sp. can attach underneath the scales of fish which could impact treatment efficacy in aquaculture.
